# P281: Adequacy to the use of personal protective equipment in the isolations of emergency

**DOI:** 10.1186/2047-2994-2-S1-P281

**Published:** 2013-06-20

**Authors:** RS Martins, NJF Cavalcante, S Scota, ADS Gomes, PB dos Santos, SR Moura

**Affiliations:** 1 Instituto de Infectologia Emilio Ribas, São Paulo, Brazil

## Introduction

A growing number of TB cases diagnosed and treated in hospitals, mainly by the association of TB with HIV infection and other immunosuppressive diseases, which poses a risk to health professionals[1]. Thus, they must be prepared to identify suspects and TB patients as early as possible, valuing aspects of prevention and use of security measures.

## Objectives

To assess the suitability for the use of personal protective equipment (PPE) by health care professionals of an Emergency Unit in accordance with the classification TSN (classification made in the hospital where the study was done for T being patient diagnosed with tuberculosis, S suspected tuberculosis and N not tuberculosis) caring for patients with suspected or confirmed tuberculosis as well as the adequacy of isolation prescribed and practiced.

## Methods

An observational study conducted at the Emergency Unit in a reference hospital for infectious diseases. The professionals were evaluated according three criteria: correct use of PPE; proper fit of PPE in the face ("Seal check"); EPI and conditioning. It was conducted an evaluation of the adequacy of isolation, by comparing what was prescribed and practiced in door / bedside.

## Results

It was evaluated 82 prescriptions, having prevailed standard precaution (47.5%), isolation 4N95 (Respiratory aerosol) (28.0%) and the classifications N (58.5%) and S + (13.4%). Of 73 observations, there was N95 mask noncompliance of 15.1%. Already in isolation 3N95 (respiratory aerosol+contact), non-adherence to this EPI was 26.67%. Regarding the use of apron and glove at isolation 3N95, nonadherence was 66.6% and 50%, respectively. From 15 observations on the implementation of the "Seal check", 40% were screened. And 30 observations against the conditioning mask N95, 86% were conditions PPE properly.

## Conclusion

There was adequacy of TSN isolation; It was observed a highest frequency of use of N95 mask in isolation 4N95 then in 3N95; less than 50% had the "seal check" and over 80% conditioned the N95 mask properly.

## Competing interests

None declared
